# Pathogenesis of Cutaneous Mycobacterial Infections—*M. marinum* and *M. leprae*

**DOI:** 10.3390/ijms26188897

**Published:** 2025-09-12

**Authors:** William Dela Cruz, Erika Fernau, Vishwanath Venketaraman

**Affiliations:** 1 College of Osteopathic Medicine of the Pacific, Western University of Health Sciences, Pomona, CA 91766, USA; william.delacruz@westernu.edu; 2 College of Medicine, Drexel University, Philadelphia, PA 19104, USA; emf322@drexel.edu

**Keywords:** *Mycobacterium marinum*, *Mycobacterium leprae*, oxidative stress, glutathione, ROS, zoonotic mycobacteria, redox imbalances

## Abstract

*Mycobacterium marinum* and *Mycobacterium leprae* are zoonotic mycobacteria causing chronic cutaneous infections that challenge host immunity and tissue integrity. Reactive oxygen species (ROS) play a complex role in the host defense system. While essential for pathogen elimination and intracellular signaling, excessive ROS can lead to immune dysregulation and impaired tissue healing. This review explores *M. marinum* and *M. leprae* pathogenesis through the role of ROS in redox imbalances, immunity, and cutaneous wound healing. Physiological ROS levels are vital for T-cell activation and differentiation. However, excessive ROS production, particularly in innate immune cells, can lead to T-cell suppression. *M. leprae* infection is associated with a significant reduction in key antioxidants such as glutathione (GSH), GSH peroxidase (GSH-Px), and GSH reductase (GR), a reduction that correlates with disease severity. For *M. marinum*, disrupting the pathogen’s redox balance through thioredoxin reductase (TrxR) inhibition sensitizes bacteria to ROS damage, reducing bacterial load. Overall, redox imbalance is central to the pathogenesis and persistence of cutaneous mycobacterial infections, compromising host defense and impairing tissue repair. Restoring and maintaining proper redox homeostasis, potentially by exploring the role of GSH as an antioxidant, represents a promising adjunct treatment to improve host outcomes in these challenging dermatological conditions.

## 1. Introduction

There are many members of the genus *Mycobacterium*. Generally, they are aerobic, non-motile, non-spore forming, Gram-positive bacilli with cell walls containing mycolic acid. This unique structure makes them acid fast on Ziehl-Neelsen staining [1]. The focus of this article will be on two species. *Mycobacterium marinum* and *Mycobacterium leprae* are slow-growing mycobacteria that can cause chronic cutaneous infections in humans, with an increasing recognition of their zoonotic potential. *M. marinum* infections are rare and are often difficult to diagnose due to long incubation periods and symptoms that resemble other skin conditions [2]. *M. leprae* presents with similar challenges. While more prevalent than *M. marinum*, diagnosing leprosy among the many differential diagnoses can be hard. The clinical diagnosis is also dependent on the recognition of disease signs and symptoms often resulting in delayed or a missed diagnosis [3]. Given their clinical similarities, diagnostic challenges, and the potential for under recognition, drawing parallels and advancing our understanding of cutaneous mycobacterial infections is crucial.

### 1.1. Mycobacterium marinum

*M. marinum* is a nontuberculous, Gram-positive, acid-fast bacillus endemic to aquatic environments, including aquariums, fish tanks, swimming pools, and natural freshwater and saltwater bodies. It is an established fish pathogen and transmission to humans typically occurs when contaminated water or aquatic animals come into contact with minor skin trauma. This leads to granulomatous cutaneous lesions often referred to as “fish tank granuloma” [4,5].

Cutaneous infection most often begins as a solitary erythematous or violaceous papule or nodule at the site of inoculation, typically on the hands or upper extremities. Over time, these lesions may ulcerate, form crusts, or take on a verrucous appearance. In some cases, lesions may spread proximally along lymphatic channels in a sporotrichoid pattern. While the disease is generally limited to the skin and subcutaneous tissues in immunocompetent individuals, more invasive manifestations, such as tenosynovitis, septic arthritis, and osteomyelitis, may develop, especially with delayed diagnosis or in immunocompromised hosts [6,7].

Diagnosing this infection can be challenging. The infection often progresses slowly and may mimic other granulomatous diseases. Culturing this bacterium requires incubation at cooler temperatures (32 °C), and biopsy specimens typically reveal granulomatous inflammation with acid-fast bacilli. The prognosis is generally favorable with timely recognition and prolonged antibiotic therapy. However, recurrence may happen if treatment is interrupted. More advanced cases that affect the joints or bone may necessitate surgical debridement in addition to the standard antimicrobial treatment. Timely diagnosis is critical because delayed recognition increases the risk of deeper tissue involvement and long-term impairment [4,5,6].

### 1.2. Mycobacterium leprae

**Figure 1:**
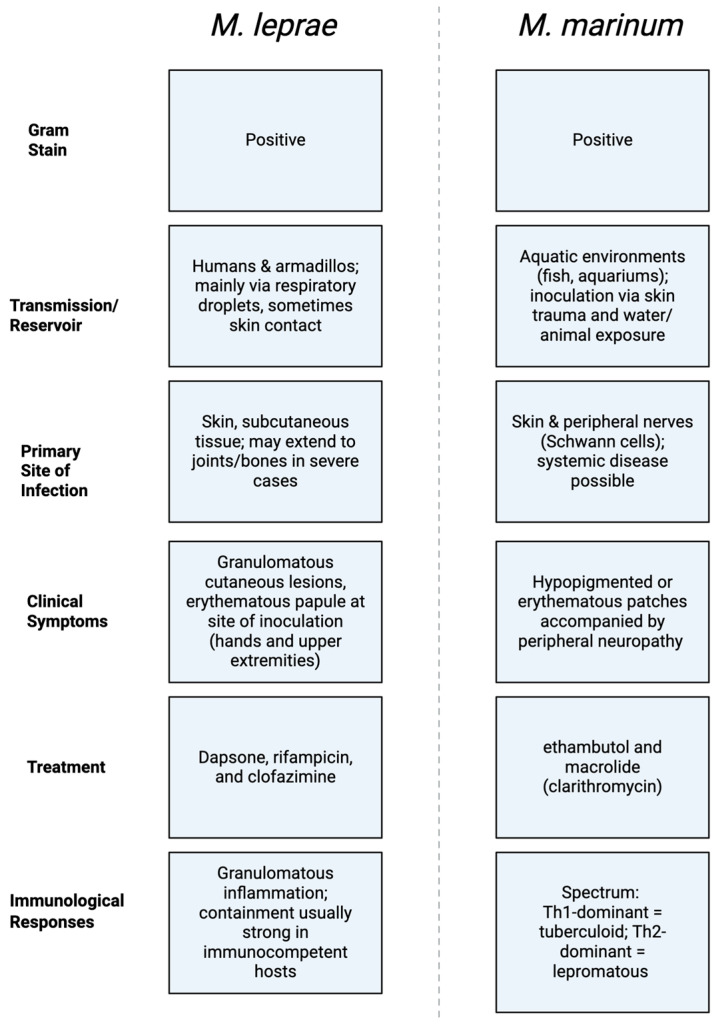
*M. leprae’s* and *M. marinum’s* Gram-stain, Transmission/Reservoir, Primary Site of Infection, Clinical Symptoms, Treatment, and Immunological Responses.

*M. leprae* is a slow growing, obligate intracellular pathogen responsible for leprosy also known as Hansen’s disease. Unlike *M. marinum, M. leprae* is primarily maintained within human and certain animal reservoirs, notably armadillos in North America. A comparison of traits between *M. leprae* and *M. marinum* is represented below in Figure 1. Transmission occurs mainly through respiratory droplets, although skin contact may also contribute in some cases [8,9]. *M. leprae* has an affinity for skin and peripheral nerves, leading to a spectrum of clinical presentations from tuberculoid to lepromatous leprosy, depending on the host’s immune response [9,10]. Cutaneous signs include hypopigmented or erythematous patches, often accompanied by sensory loss. Nodules and diffuse skin thickening may also be observed. Nerve involvement results in sensory deficits and muscle weakness, with potential for deformities if untreated [11,12].

In addition to the dermatologic manifestations, nerve involvement is a key feature of disease progression. The infection of Schwann cells leads to peripheral neuropathy, resulting in symptoms such as numbness and muscle weakness. If left untreated, this can cause significant disability due to motor impairment and deformities. The degree of nerve damage often correlates with the specific form of the disease; for instance, patients with tuberculoid leprosy may experience localized nerve impairment, whereas those with lepromatous disease typically present with widespread and symmetric nerve involvement. Chronic inflammation and repeated trauma to anesthetic areas may lead to secondary injuries, ulcers, and erosion of the bone in the digits [2,8,10].

Diagnostic procedures rely on a combination of clinical assessment and laboratory confirmation. Skin smears and biopsies may detect acid-fast bacilli, while histopathological analysis frequently exhibits granulomatous inflammation with varying bacterial loads, dependent on the type of disease. Polymerase chain reaction (PCR) can detect *M. leprae* DNA in cases that are negative on traditional skin smears. While PCR is highly sensitive for multibacillary cases, its use is limited in many endemic regions due to high cost and laboratory requirements [2].

The prognosis is significantly influenced by the promptness of diagnosis and the initiation of treatment. Early intervention with multidrug regimens can halt disease progression and prevent disability, whereas delayed recognition heightens the risk of irreversible nerve damage and lasting impairment. Despite the drastic reduction in prevalence worldwide due to multidrug therapy, untreated or late-treated cases continue to pose a substantial cause of morbidity in endemic regions [2,10,11].

## 2. Methods

We conducted a comprehensive literature review to examine the pathogenesis of cutaneous infections caused by *M. marinum* and *M. leprae,* with a focus on the role of oxidative stress and redox imbalance in host–pathogen interactions. Relevant studies were identified through a systematic search of PubMed and Google Scholar databases up to July 2025. We included peer-reviewed research articles, systematic reviews, and case reports. Articles not published in English, were excluded. The findings from the selected studies was organized into thematic sections to highlight the shared and distinct mechanism underlying disease pathogenesis.

## 3. Entry, Invasion, and Tropism

*M. marinum* and *M. leprae* both have a predilection for infecting cooler regions of the body, especially the skin and peripheral nerves. Both organisms grow optimally at temperatures lower than core body temperature. *M. marinum* typically gains entry through minor abrasions or puncture wounds following exposure to contaminated aquatic environments. The organism invades the dermis and subcutaneous tissues, where it replicates within macrophages. This often leads to granulomatous inflammation and the development of nodular skin [6,13]. Similarly, *M. leprae* enters the body via respiratory droplets or through broken skin after prolonged contact with infected individuals or zoonotic reservoirs. Once inside the host, *M. leprae* targets Schwann cells and macrophages in the cooler areas of the body, such as the skin, nose, and peripheral nerves. This skin tropism may be due to the bacteria’s inability to thrive at higher core body temperatures and its interactions with localized immune responses. In both infections, the skin serves as a permissive environment, where macrophage-mediated immunity attempts to contain the infection; however, the pathogens’ immune evasion strategies allow them to persist and cause chronic disease [8,11].

## 4. Oxidative Stress in Cutaneous Mycobacterial Infection

### 4.1. Reactive Oxygen Species and Host Defenses

As the body encounters invading pathogens such as bacteria, Reactive oxygen species (ROS) play a crucial role in the host’s innate immune response. Phagocytic cells, including neutrophils and macrophages detect microbial components such as lipoproteins primarily through recognitions by toll-like receptors. This recognition triggers the host immune system and stimulates the production of ROS [14,15].

Phagocytes contain a membrane-associated NADPH oxidase complex, that when activated initiates the process known as respiratory burst and the subsequent production of ROS [16,17]. During this process, phosphorylation of the p47^phox^ subunit causes it to undergo conformational change allowing it to interact with p22^phox^. This interaction leads to the assembly of the complete oxidase complex. This complex facilitates the transport of electrons from cytoplasmic NADPH to molecular oxygen, generating the reactive oxygen species superoxide (O_2_^−^). O_2_^−^ serves as a precursor to more reactive ROS such as hydrogen peroxide (H_2_O_2_) and singlet oxygen (^1^O_2_). These ROS are essential for the intracellular killing of engulfed pathogen [18].

These ROS exert antimicrobial activity through multiple mechanisms. Oxidative stress induced by ROS can damage microbial DNA through strand breaks or base modification. Additionally, ROS can disrupt lipid membranes through a process known as lipid peroxidation. The oxidation of proteins can lead to inactivation of key bacterial enzymes. Collectively, ROS can compromise pathogen’s structural integrity and function, as well as facilitate the clearance of the pathogen by the host immune system [19,20,21].

### 4.2. ROS and Immune Modulation

ROS are not merely destructive agents; they also act as crucial intracellular messengers that guide immune cell decisions. While often associated with damage, physiological concentrations of ROS serve as vital signaling molecules that can shape the adaptive immune response, rather than solely acting as antimicrobials [22,23].

T cells, which are critical to the adaptive immune response, exemplify this intricate regulation. When an antigen stimulates the T-cell receptor (TCR) and CD80/CD86 binds to CD28, T cells secrete cytokines like interleukin-2 (IL-2). This induces the activation of transcription factor Akt. Akt then initiates the mTOR pathway, causing a shift in cellular energy metabolism from oxidative phosphorylation to glycolysis. The increase in nutrient uptake and glucose metabolism promotes the proliferation of naïve T cells. While CD4+ T-cell differentiation depends largely on aerobic glycolysis, specific subsets like regulatory T cells rely on fatty acid oxidation instead [24,25].

ROS, as byproducts of oxidative metabolism rapidly increase during T-cell activation due to heightened energy demands. ROS is produced from mitochondrial Complexes I, Complex III, and NADPH oxidase. Specifically, medium or low concentrations of ROS act as intracellular signaling molecules, regulating TCR signaling components such as Lck and ZAP-70 [25,26]. They modulate transcription factors like NF-κB, AP-1, and NFAT via MAPK pathways whose enzymes such as Erk, JNK, P38 phosphorylation depends on H_2_O_2_. Through the inhibition of mitochondrial Complex I and Complex III, T-cell activation and cytokine secretion was significantly impaired. Thus, ROS production upon TCR stimulation promotes broad TCR signaling and transcriptional activation of IL-2, underscoring their critical role in T-cell activation [25,27,28].

However, the beneficial role of ROS is concentration-dependent, as excessive intracellular or extracellular ROS production in innate immune cells, including monocytes, macrophages, and dendritic cells (DCs), can lead to detrimental effects. While such ROS production is associated with antigen presenting cell formation and activation, it ultimately leads to T cell suppression, partially mediated by the upregulation of the co-inhibitory immune checkpoint molecule, programmed death ligand 1 (PD-L1). This shift results in macrophages acquiring an immunosuppressive phenotype and secreting immunosuppressive cytokines, often in response to ROS inducers [29,30,31].

### 4.3. Immune Suppression in M. leprae Infection

This T cell suppression observed with excessive ROS, is critical in *M. leprae* infection. Analysis of human peripheral blood tissue in *M. leprae* cases shows a downregulation of antigen presentation via MHC I and II. This indicates that *M. leprae* infected host dendritic cells and macrophages reduce the stimulation of both CD4+ and CD8+ T cells. Reduced T cell stimulation compromises the host defense, by reducing interferon-gamma secreted by Th1 CD4+ and cytotoxic T cells. Additionally, a decrease in crucial proinflammatory cytokines such as IL-6, tumor necrosis factor alpha (TNF-α), and IL-1β was also observed [32,33].

**Figure 2:**
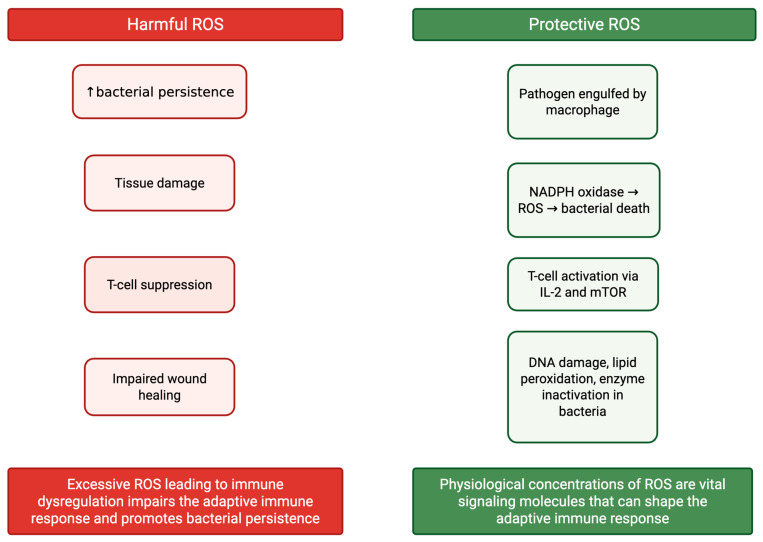
The dual role of ROS in cutaneous mycobacterial infections. At physiological levels, ROS promote immune activation and bacterial killing. Excessive ROS production, however, suppresses adaptive immunity and contributes to tissue damage and chronic infection.

Therefore, excessive ROS leading to immune dysregulation impairs the adaptive immune response and promotes bacterial persistence. This underscores the critical need for redox balance, which the immune system relies on antioxidant systems to maintain. The dual role of ROS, shown in Figure 2, as both a defense against infection and a mediator of tissue damage highlights the importance of redox homeostasis for a healthy and successful immune response [34]. Disruption of this balance can significantly impair pathogen clearance, making it a compelling target for therapeutic intervention.

### 4.4. Redox Imbalance in M. leprae

In leprosy, phagocyte activation leads to production of ROS. While intended to control infection, this can lead to collateral damage to host tissues through lipid peroxidation. The antioxidant glutathione (GSH) plays a crucial role in protecting the cells and neutralizing excessive ROS [35,36].

One study assessed the blood GSH content in 100 patients with leprosy and compared them to 50 healthy controls. GSH levels were measured and estimated using the DTNB [5,5′-dithiobis (2-nitrobenzoic acid)] reduction method. Enzyme activity of GSH-Px and GR were measured in the red blood cell hemolysate by kinetic methods. The results showed that GSH, glutathione peroxidase (GSH-Px), and glutathione reductase (GR) were significantly lower in leprosy patients. The study also noted a progressive decreasing trend in GSH levels and enzyme activity with increasing levels of bacteriological index. This suggests that *M. leprae* infection leads to oxidative stress associated with diminished antioxidant defense potential, with more profound deficits seen towards the lepromatous end of the disease spectrum. Reduced antioxidant potential appears to be associated with both bacterial load and clinical type of leprosy, highlighting the key role that redox imbalance plays in diseases severity and progression [37].

### 4.5. Redox Imbalances in M. marinum

In early stages of *M. marinum* infection, neutrophils provide crucial protection by killing bacteria through NADPH-dependent oxidative bursts. Individuals with reduced levels of NADPH-dependent oxidative burst mechanism, have shown higher susceptibility to mycobacterial infections, highlighting the significant role of ROS in innate immunity from *M. marinum* infection [38].

As infection progresses, excess tumor necrosis factor (TNF) was seen in the zebrafish models to stimulate ROS production and activate Cyclophilin D, a mitochondrial permeability transition pore regulator, triggering programmed necrosis of infected macrophages. This cascade of mitochondrial ROS production led to the activation of lysosomal enzyme and Bel-2-associated X protein (BAX), subsequently stimulating endoplasmic reticulum (ER) ryanodine receptors to cause a flow of calcium into the mitochondrion. This calcium overload causes Cyclophilin D-mediated necrosis [39,40]. Although necrosis helps eliminate infected immune cells, it may paradoxically release intracellular bacteria, potentially worsening infection outcomes through further inflammation and dissemination of the pathogen [39,41].

*M. marinum* also relies on thioredoxin reductase (TrxR) to maintain redox balance for its own survival. Ebselen, which mimics GSH-Px, targets bacterial TrxR. TrxR activity was measured using 5,5’-dithiobis-(2-nitrobenzoic acid) (DTNB) assay to assess the effects on bacterial redox homeostasis. By inhibiting TrxR in mouse models affected with *M. marinum*, ebselen combined with streptomycin significantly increased cellular oxidative stress, leading to bacterial cell death, impaired proliferation, and ultimately rescued the mice from high bacterial load. The critical function of ROS as a virulence factor was confirmed by dithiothreitol (DTT), a reducing agent, which reversed the ebselen induced harm [42,43,44].

The importance of ROS in controlling *M. marinum* is further magnified by the bacterium’s intrinsic defense mechanisms. The *M. marinum* mycobacterial enhanced locus 2 (mel2), with genes showing similarities to bacterial bioluminescence systems, plays a crucial role in defending against ROS and reactive nitrogen species. A mutation in the mycobacterial enhanced locus F (melF) gene, for instance, leads to increased susceptibility to these reactive species, indicating a previously unrecognized pathway for bacterial resistance and highlighting the pathogen’s vulnerability when its defenses are compromised [45,46].

These findings highlight a distinct pathogenic strategy where redox manipulation is important not only for the pathogenesis of *M. marinum* but also a key target to reducing bacterial load. However, there is no current evidence linking a baseline reduction in GSH and its enzymes to *M. marinum* infections as seen in *M. leprae*. Still there is a possibility to look at balancing GSH levels to treat the condition.

## 5. Oxidative Stress and the Skin

### 5.1. ROS and Skin Aging

Aging, as manifested on the skin, results in significant changes in its structure, function, and appearance. ROS are associated with a core mechanism mediating skin aging, leading to lipid, protein, nucleic acid, and organelles damage. Many studies assess the effects of ultraviolet radiation, where the underlying mechanism involves an increase in ROS damaging skin structure, skin function, and mediating inflammatory responses [47,48,49]. ROS facilitates activation of pathways that reduce collagen production and synthesis, activation of metalloproteinases responsible for degrading connective tissues, and induces the secretion of senescence-associated secretory phenotype, ultimately promoting aging of skin [48,50].

### 5.2. ROS on Skin Wound Healing

Beyond general aging, localized oxidative stress significantly impairs wound healing. When assessing foot ulcers due to diabetes mellitus, there are many factors that may play a role. However, there is a high influence and association to oxidative stress such that imbalance in free radicals and antioxidants, through the overproduction ROS can delay wound healing [51]. Nonhealing diabetic wounds are infiltrated by highly oxidizing environments associated with hyperglycemia and tissue hypoxia, directly contributing to this delay in skin injury repair. This suggests that high oxidative stress levels predispose wounds to poor healing, lesion persistence, and chronic disease progression [51,52].

Recognizing this, in addition to antimicrobial treatment, decreasing ROS to reduce oxidative stress and improve healing are crucial targets in the care of *M. leprae* and *M. marinum*, both of which present with cutaneous lesions. As such, GSH, capable of reducing ROS levels, represents a promising adjunct therapy to decrease chronic oxidative damage and improve skin health.

## 6. Glutathione

### 6.1. Glutathione Role in Redox Balance

GSH is essential for maintaining redox homeostasis and removing ROS. GSH’s ability to be easily oxidized and dehydrogenated through its thiol group, allows it to effectively scavenge and eliminate free radicals. Furthermore, GSH also provides essential electrons for enzymes like GSH-Px, which reduce the level of ROS by facilitating H_2_O_2_ to water [53]. Through these mechanisms, GSH is vital in maintaining intracellular redox balance and mitigating oxidative damage [54].

### 6.2. Glutathione in Immune Regulation

**Figure 3:**
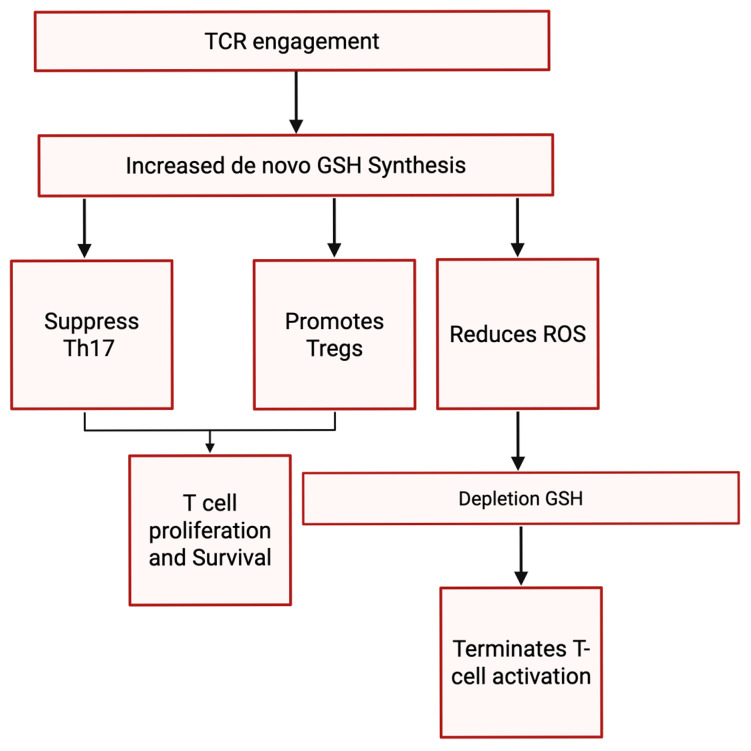
Illustrates the signaling pathway involved in TCR engagement leading to increased synthesis of GSH. It highlights how GSH synthesis suppression of Th17 cells and promotion of Tregs occurs, alongside a reduction in ROS. Subsequently, T cell proliferation and survival are stimulated, while the depletion of GSH results in the termination of T cell activation.

This crucial role in redox control becomes particularly significant during immune responses. As ROS levels increase during T-cell receptor (TCR) engagement, GSH synthesis also increases. This heightened GSH production works to counteract the potentially detrimental effects of excessive ROS, thereby significantly impacting T-cell survival and function. Notably, this increased de novo GSH synthesis actively suppresses Th17 differentiation while simultaneously promoting the production of regulatory T cells (Tregs). Conversely, the depletion of GSH or the loss of its de novo synthesis under chronic stress conditions compromises vital metabolic pathways regulated by mTOR, NFAT, and N-Myc, ultimately leading to the termination of T-cell activation as shown in Figure 3 [55].

### 6.3. Glutathione’s Role in Mycobacteria Infection

Addressing the reduction in GSH in infections, one study highlighted its effects on *M. tuberculosis* infection. N-acetyl cysteine, a GSH precursor, was shown to improve control of intracellular *M. tuberculosis* infection by decreasing the levels of IL-1, TNF-a, and IL-6 [56]. While *M. tuberculosis* is primarily human–human transmission, unlike the zoonotic potential of *M. marinum* and *M. leprae*, there are still commonality in how redox balance plays a role in pathogenicity of these diseases. Another study demonstrated that the topical application of a glutathione-cyclodextrin nanoparticle complex conferred therapeutic benefits against *M. avium* by enhancing the local immune response. To date, glutathione has been administered topically, orally, and parenterally in the management of skin conditions. Its most common use has focused on cosmetic purposes such as skin whitening and preventing wrinkles. However, the clinical application of glutathione orally via its precursor N-acetyl cysteine or topically through nanoparticle formulations have been shown to be effective in certain mycobacterium. This warrants consideration of its potential role in infections caused by *M. leprae* and *M. marinum*. Clinical evidence for these approaches remains limited and future research is required to establish their efficacy and optimal delivery routes [57,58].

## 7. Current Treatments

### 7.1. Treatment of M. leprae

The treatment of leprosy caused by *M. leprae* follows World Health Organization multidrug therapy guidelines, which remain the global standard. Multidrug therapy typically includes a combination of dapsone, rifampicin, and clofazimine, chosen to prevent resistance and improve therapeutic outcomes. Dapsone, a bacteriostatic sulfone antibiotic, inhibits folate synthesis but may lead to hemolytic anemia, particularly in patients with glucose-6-phosphate dehydrogenase deficiency. Rifampicin is highly bactericidal and rapidly decreases the bacillary load, while clofazimine is both anti-inflammatory and weakly bactericidal, making it effective in managing multibacillary disease and lepra reactions [59,60].

Treatment duration is guided by bacillary burden. Paucibacillary leprosy requires 6 months of dapsone and rifampicin, while multibacillary disease is treated for 12 months with all three drugs. Clinical monitoring is needed due to the potential for medication toxicity and immune reactions such as erythema nodosum leprosum. Despite the chronic nature of leprosy infection, it is curable with prompt and consistent treatment, and multi-drug therapy has dramatically reduced the global disease burden [61,62].

Erythema nodosum leprosum (ENL) is an immunological complication that affects many patients with lepromatous leprosy. It presents as painful nodular skin lesions and can also involve nerves, joints, and other organs. ENL may occur before, during, or after the completion of multidrug therapy. Managing this condition often requires prolonged corticosteroid treatment, which can lead to serious side effects and poses challenges, especially in resource-limited settings. Timely recognition of ENL is crucial to prevent permanent nerve damage and disability, and it is essential for patients to comply with treatment for successful outcomes [63].

Adherence to prolonged therapy can be challenging, especially in regions with limited healthcare infrastructure. To enhance compliance, the WHO provides free multidrug therapy in endemic countries. Although resistance to dapsone or rifampicin is rare, it can occur with incomplete or interrupted treatment. HIV-positive patients may experience altered immune responses, potentially affecting the progression of the disease and the management of leprosy reactions. Ultimately, the success of treatment depends on patient education, adherence to the treatment plan, and the early recognition of inflammatory complications [58,59].

### 7.2. Treatment of M. marinum

In contrast, management of *M. marinum* infections typically involves combination antimicrobial therapy for extended durations. The most frequently used regimen includes ethambutol plus a macrolide, such as clarithromycin or azithromycin. This approach has high cure rates and a favorable tolerability profile. Monotherapy, especially with doxycycline, may be considered in mild, localized cases [62,64].

Treatment duration typically extends for 1 to 2 months beyond clinical resolution, often totaling 3 to 6 months. For deeper and more invasive infections, surgical debridement may be necessary in addition to antibiotic therapy. It is crucial to adhere closely to the prolonged therapy regimens, as premature discontinuation can lead to relapse or treatment failure. Resistance to tetracyclines has been reported which suggests caution when using doxycycline or minocycline as monotherapy. This highlights the potential value of susceptibility testing to guide therapy. Patients with immunocompromising conditions, such as HIV infection or those using immunosuppressive medications, may require longer courses of treatment and closer monitoring due to an increased risk of disease dissemination. Additionally, drug tolerability and potential adverse effects should be taken into account. For example, ethambutol can cause optic neuropathy while macrolides can cause gastrointestinal upset. Early recognition of infections is crucial to prevent progression to deeper tissue involvement and to allow for the timely initiation of therapy [64,65].

## 8. Discussion

This comprehensive review highlights the critical role redox imbalance plays in the pathogenicity of *Mycobacterium marinum* and *Mycobacterium leprae* cutaneous infections. Within both *M. leprae* and *M. marinum* infection, the host immune functions are driven by dysregulated ROS and compromised antioxidants. Excessive ROS production in innate immune cells suppresses T-cell activity though increased levels of PD-L1, ultimately leading to a compromised host defense. The presence of persistent lesions in these infections is partly attributable to the negative effects of imbalanced ROS on cutaneous wound healing. Evidence from *M. leprae* infections indicates a direct link to reduced host GSH levels and its related enzymes, correlating with disease severity. While *M. marinum* has yet to be studied to prove a baseline reduction in host GSH during active infection, mutating the *M. marinum* mel2 locus reduced the pathogen ability to defend itself against ROS proving significance in pathogen survival. GSH’s multifaceted role in directly neutralizing ROS and modulating immune responses counteract these observed pathologies and enhances host ability to clear infection and repair tissue. These findings fuel a growing interest in host-directed therapies. Focusing on redox balance offers an approach applicable to chronic or inflammatory dermatological conditions characterized by oxidative stress, moving beyond sole reliance on antimicrobial agents. Effectively managing chronic infections as seen in *M. marinum* and *M. leprae* necessitates addressing not only the pathogen but also the host’s capacity to mount a balanced immune response and facilitate tissue repair. Redox modulation offers a pathway to enhance host resilience and the skin’s ability to heal. Current antimicrobial treatments, despite reducing bacterial load, often involve lengthy regimens and do not fully prevent lesion persistence, underscoring the clear need for adjunct therapies that address the underlying host environment and its immune dysregulation. Future research should prioritize conducting in-depth preclinical studies in animal models of *M. marinum* and *M. leprae* skin infection, followed by clinical trials to evaluate the efficacy, optimal dosing, and appropriate delivery methods of GSH as a therapeutic option. Additionally, exploring potential synergistic effects of GSH when combined with existing antimicrobial regimens and other host-directed therapies is crucial, as is expanding research to elucidate specific redox profiles and mechanisms of imbalance, particularly given the limited data on direct links to reduced GSH levels in *M. marinum* infections.

## Abbreviations

GSH	Glutathione
ROS	Reactive Oxygen Species
H_2_O_2_	Hydrogen peroxide
TCR	T-cell Receptor
IL	Interleukin
DCs	Dendritic cells
PD-L1	Programmed Death Ligand 1
GSH-Px	Glutathione Peroxidase
GR	Glutathione Reductase
TNF	Tumor Necrosis Factor
TrxR	Thioredoxin Reductase
Tregs	Regulatory T cells
